# Case Report: The Importance of Novel Coronavirus Disease (COVID-19) and Coinfection with Other Respiratory Pathogens in the Current Pandemic

**DOI:** 10.4269/ajtmh.20-0266

**Published:** 2020-04-17

**Authors:** Karam Khaddour, Anna Sikora, Nayha Tahir, Daniel Nepomuceno, Tian Huang

**Affiliations:** 1 Department of Internal Medicine, Rosalind Franklin University of Medicine and Science, North Chicago, Illinois;; 2 Department of Intensive Care, Northwestern Medicine McHenry Hospital, McHenry, Illinois;; 3 Metro Infectious Disease, Northwestern Medicine McHenry Hospital, McHenry, Illinois

## Abstract

The early shortage of novel coronavirus disease (COVID-19) tests in the United States led many hospitals to first screen for common respiratory pathogens, and only if this screen was negative to proceed with COVID-19 testing. We report a case of a 56-year-old woman with severe acute respiratory syndrome-coronavirus-2 (SARS-CoV-2) coinfection with group A *Streptococcus*. The initial testing strategy resulted in delays in both diagnosis and implementation of appropriate precautions. Underlined is the importance of testing for both SARS-CoV-2 and other common respiratory pathogens during the current pandemic.

## INTRODUCTION

The recent outbreak of the novel coronavirus called severe acute respiratory syndrome-coronavirus-2 (SARS-COV-2) was declared a pandemic on March 11, 2020 by the WHO.^1^ The current main modality of testing is real-time reverse transcription–polymerase chain reaction (RT-PCR).^2,3^ The early shortage of available tests in the United States led many healthcare facilities to ration tests and adopt an algorithmic approach for testing. These algorithms included initial testing for common respiratory pathogens, and if negative, proceeding with novel coronavirus disease (COVID-19) testing if there was a strong suspicion. Here, we present a case of coinfection of a common respiratory pathogen and SARS-COV-2. A review of other case reports demonstrating coinfection with SARS-COV-2 is included. Based on these findings, we recommend testing for SARS-COV-2 and other common respiratory pathogens to ensure accurate diagnosis, prompt patient management, and appropriate isolation.

## CASE

**Figure 1.**
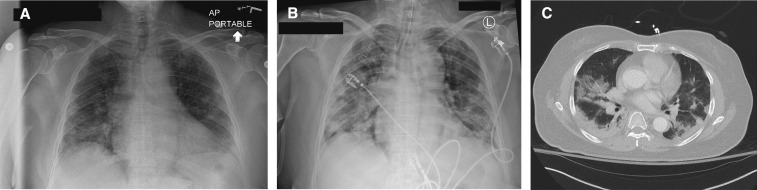
Chest imaging studies. (**A**) Chest X-ray on the first day of admission showing small scattered patchy airspace opacities bilaterally. (**B**) Chest X-ray on the second day of admission showing increased patchy infiltrates bilaterally. (**C**) Chest computed tomography showing scattered peripheral and predominantly dependent patchy airspace consolidation with ground-glass opacities.

A 56-year-old female presented to the hospital with complaints of fever, sore throat, dry cough, and myalgia for 2 weeks. Past medical history was significant for hypertension, treated with amlodipine and benazepril, and chronic back pain. She had no recent travel history or known ill contacts. Chest X-ray showed scattered patchy airspace opacities bilaterally (Figure 1A). Viral influenza A and B PCR tests were negative. Rapid *Streptococcus* A antigen testing was positive. She was treated with ceftriaxone and azithromycin. Repeat chest X-ray the next day (Figure 1B) revealed worsening bilateral diffuse opacities. Given persistent hypoxia and fever, a respiratory virus panel (Respiratory Pathogen Panel- NAT/Quest Diagnostics, Valencia, CA) was sent and returned negative. Subsequently, the patient had worsening of symptoms within 24 hours and underwent a SARS-CoV-2 rRT-PCR, and the test was positive. CT of the chest showed scattered perihilar and dependent patchy airspace consolidation and bilateral ground-glass opacities (Figure 1C). The patient was subsequently intubated and mechanically ventilated during the second day of hospitalization, given her refractory hypoxia and development of acute respiratory distress syndrome. The patient was started on hydroxychloroquine sulfate and tocilizumab on the second day of admission. On the fourth day of admission, the patient remained hypoxic without signs of multiorgan damage. She was started on extracorporeal membranous oxygenation (ECMO) for 1 week, and then later taken off ECMO in stable condition. Three weeks after admission, the patient was extubated and discharged to inpatient rehabilitation.

## DISCUSSION

Despite an increase in the number of confirmed COVID-19 cases in the United States, there is currently still limited capacity for viral testing, which can hinder identification of patients who might benefit from early treatment and interventions, and who require isolation.^4^ Given the early shortage of tests, many hospitals adopted protocols to limit testing to highly suspected cases. One of these measures was to screen for common viral and bacterial pathogens in patients who present with respiratory symptoms before proceeding with SARS-CoV-2 laboratory testing. Based on this approach, if a pathogen other than SARS-CoV-2 is identified, standard practice has been not to proceed with SARS-CoV-2 testing. However, early experience and the recent literature have shown that coinfection with SARS-CoV-2 and other respiratory pathogens can be fairly common.^5^ A recent study showed that five of 115 patients infected with SARS-COV-2 were coinfected with influenza virus (rate of 4.35%).^6^ In another study, of 21 patients infected with SARS-COV-2, two tested positive for influenza A and one positive for parainfluenza type-3.^7^ Other case reports have described coinfection with other respiratory pathogens (Table 1).

**Table 1:** Review of literature describing cases of novel coronavirus disease coinfection with other respiratory pathogens and the outcomes

Authors and reference	Country	Number of coinfected patients	Age (years)	Gender	Coinfecting respiratory pathogen	Complications	Outcome
Ding et al.^6^	China	5	Mean 50	2 Males and 3 females	3 Influenza A and 2 influenza B	1 ARDS,* 3 abnormal liver function, and 2 diarrhea	5 Hospitalized, not requiring ICU,† and five discharged
Arentz et al.^7^	United States	3	Not available	Not available	2 Influenza A and 1 parainfluemza type 3	Not available	Not available
Fan et al.^8^	Singapore	1	36	Male	*Mycoplasma pneumoniae*	Cold agglutinin and rouleaux formation without hemolytic anemia	ICU† admission
Wu et al.^9^	China	1	69	Male	Influenza A	ARDS*	Not available
Touzard-Romo et al.^10^	United States	1	Not available	Not available	Metapneumovirus	Not available	Not available

* ARDS–acute respiratory distress syndrome.

† ICU–intensive care unit.

The findings of coinfection with different respiratory pathogens and SARS-CoV-2 challenge the use of an algorithm requiring prior testing for viral respiratory and bacterial pathogens before testing for SARS-COV-2. Undiagnosed COVID-19 results in inadequate isolation that can affect patient care and place healthcare workers at risk of contracting the virus. In the current worldwide pandemic, physicians and healthcare workers should have a high index of suspicion to test for SARS-CoV-2 in patients presenting with symptoms compatible with COVID-19 even when they test positive for other respiratory pathogens.
